# A new clustering model based on the seminal plasma/serum ratios of multiple trace element concentrations in male patients with subfertility

**DOI:** 10.1002/rmb2.12584

**Published:** 2024-05-28

**Authors:** Takazo Tanaka, Kosuke Kojo, Yoshiyuki Nagumo, Atsushi Ikeda, Takuya Shimizu, Shunsuke Fujimoto, Toshiyuki Kakinuma, Masahiro Uchida, Tomokazu Kimura, Shuya Kandori, Hiromitsu Negoro, Hiroyuki Nishiyama

**Affiliations:** ^1^ Department of Urology, Faculty of Medicine University of Tsukuba Tsukuba Ibaraki Japan; ^2^ Center for Human Reproduction International University of Health and Welfare Hospital Nasushiobara Tochigi Japan; ^3^ Health Care Analysis Center Renatech Co., Ltd. Isehara Kanagawa Japan; ^4^ Department of Urology Tsukuba Gakuen Hospital Tsukuba Ibaraki Japan

**Keywords:** accessory gland, arsenic, phosphorus, split ejaculation sampling, trace element

## Abstract

**Purpose:**

To investigate whether seminal plasma (SP)/serum ratios of multiple trace elements (TEs) can classify patients with male subfertility.

**Methods:**

SP/serum ratios of 20 TEs (lithium, sodium, magnesium, phosphorus, sulfur, potassium, calcium, manganese, iron, cobalt, copper, zinc, arsenic, selenium, rubidium, strontium, molybdenum, cesium, barium, and thallium) were calculated for healthy volunteers (*n* = 4) and those consulting for male subfertility (*n* = 245). Volunteer semen samples were collected by split ejaculation into early and subsequent fractions, and SP/serum ratio data were compared between fractions. The patients' SP/serum ratio data were used in an unsupervised clustering analysis and qualitatively compared with the data from the fractions of ejaculation from the volunteers. Semen quality parameters and pregnancy outcomes were compared between patient clusters.

**Results:**

The early fraction of volunteers was characterized by lower phosphorus and arsenic and 18 other higher TEs than the subsequent fraction. Cluster analysis classified patients into four distinct clusters, one sharing characteristics with the early fraction and another with the subsequent fraction. One cluster with the early fraction characteristics had significantly lower semen volume and higher pregnancy rates from spontaneous pregnancies or intrauterine insemination.

**Conclusions:**

Classification of patients based on SP/serum ratios of multiple TEs represents the dominance of fractions of ejaculation samples.

## Graphical abstract

A summary of the research approach for designing and explaining a new clustering model.

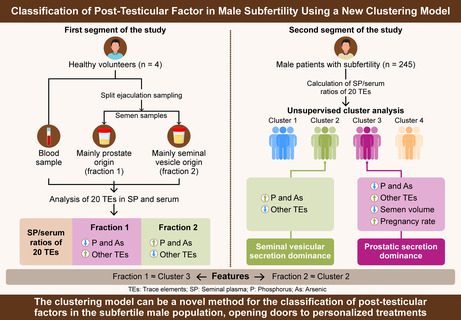

## 1 INTRODUCTION

Subfertility,^1^ which affects 12% of couples desiring children, contributes to the concerns about global population decline.^2^ In 50% of these couples, the issue primarily lies with the males, usually attributed to semen abnormalities. Semen analysis is essential for evaluating the subfertility caused by such abnormalities.^3^ However, the accuracy of conventional semen analysis is questionable, as approximately 40% of male patients with subfertility have normal sperm concentrations and motility.^4^ Moreover, most semen abnormalities stem from idiopathic causes.^3^ Empiric therapies, including supplementation, can be effective^5^; however, numerous cases remain subfertile, and there is no reliable way to decide which cases benefit from such treatments.^6^

Therefore, focusing beyond the sperm alone is important to overcome the limitations of semen analysis.^7^ Investigating the post‐testicular factors that are explained by the function of the epididymis and accessory glands is also crucial.^8^ Split ejaculation sampling is a valid technique for exploring this factor. It utilizes the fact that the first one‐third fraction of the ejaculation sample contains a dominant portion of prostatic secretion, while the subsequent two‐thirds fraction mainly originates from seminal vesicular secretion.^9^ This method enables the identification of variations in accessory gland function. For example, excessive seminal vesicular secretion can reduce sperm motility, and premature seminal vesicular emission or delayed prostatic expulsion can alter semen composition.^10^

However, the standardized procedure for semen analysis requires a thorough mixing of the sample before removing an aliquot for assessment. This process potentially obscures valuable information about the individual component.^10^ Therefore, alternative approaches are necessary to avoid the reliance on split ejaculation sampling. Each accessory gland specifically secretes some biochemical markers in seminal plasma (SP), and some types of trace elements (TEs) in SP could play a potential role as markers,^11^ with zinc (Zn) being the most robust marker reflecting prostatic secretion.^10^ Furthermore, a few reports have proposed that the ratio of SP to serum concentration differs for each substance measured, implying variations in each accessory gland secretory capacity^12^; however, no report has analyzed the simultaneous measurement of comprehensive TEs in both SP and serum.

Recent advancements in measurement techniques have enabled a comprehensive analysis of numerous TEs.^13^ Machine learning algorithms have also been used to identify potential patterns from multiple parameters.^14^ Particularly, unsupervised cluster analysis has emerged as a method that does not focus solely on apparent information but can elucidate complex interrelations among various variables.^15^ Notably, applying these sophisticated algorithms has facilitated the clustering of diverse cases of male subfertility.^16^, ^17^, ^18^ However, a clear methodology for detecting TE patterns that are associated with male subfertility has not been established.

Therefore, this study used a cross‐sectional design in two segments. In the first segment, healthy volunteers were recruited and instructed to undergo split ejaculation sampling. This process was key to reconfirming the existing knowledge regarding the differences in semen quality parameters among the early and subsequent fractions of the ejaculation samples. Multiple concentrations of TEs were measured in the SP of each fraction and serum sample. The SP/serum ratios of the multiple concentrations of TEs in the two fractions were calculated and compared to investigate the ratio characteristics of each fraction. Patients who consulted for male subfertility were recruited in the second segment of this study, and this enabled the examination of SP/serum ratios of multiple TEs in a real‐world male with a subfertility population.

This study aimed to explore the association between TEs and each fraction of the ejaculation samples and develop a new clustering model based on the SP/serum ratios of multiple TEs using unsupervised machine learning technology.

## 2 MATERIALS AND METHODS

### 2.1 Participants, clinical samples, and data selection

Four individuals who were available to undergo split ejaculation sampling and blood collection on the same day were recruited between February 2019 and March 2020 for the healthy volunteer cohort. They were selected based on the following two criteria: their partners had been pregnant and had given birth within the past 2 years, or they underwent semen analysis at least two times, with results surpassing the World Health Organization (WHO) 2010 criteria.^19^ All participants provided written informed consent. The ages of the four volunteers were 28, 29, 32, and 33 years, respectively. Among them, two volunteers had already achieved spontaneous pregnancy with their partner and had at least one child; one continued to use birth control, and the other was single. Here, the early and subsequent fractions of ejaculation samples were collected from these volunteers using a minimally modified procedure of split ejaculation sampling from previous studies.^10^ Briefly, two containers were prepared for each ejaculation; one container each was used to retrieve a fraction in the early (fraction 1) and subsequent (fraction 2) phases of ejaculation. During ejaculation, the volunteers were directed that the volume of fraction 1 should be approximately between 30% and 50% of the entire sequence of ejaculation. Each experiment was conducted at least 1 week apart and repeated at least once per volunteer for a total of 11 sessions. However, two sessions were excluded because the volume of fraction 1 was higher than that of fraction 2. After successful collection, blood samples were obtained on the same day, resulting in nine sets each of fraction 1, fraction 2, and blood samples.

Regarding the male subfertility cohort, 299 patients were recruited from among those seeking consultation for screening or the treatment of male subfertility at the International University of Health and Welfare (IUHW) Hospital in Tochigi, Japan, between August 2019 and April 2022. Participation was contingent on written informed consent. The following two inclusion criteria were applied: availability of semen aliquots for TE analyses and consent for additional blood sampling for TE level assessment. Many patients who declined blood sampling underwent endocrinological screening at other medical facilities. The sole exclusion criterion was a history or presence of malignant tumors since TEs in body fluids may be associated with cancer risk.^20^ Finally, 245 patients provided both semen and blood samples for this study (Figure S1). These patients did not practice split ejaculation sampling but instead had their SP/serum ratio data characteristics qualitatively compared with those of the healthy volunteers, as described below.

Notably, to collect practical data from a real‐world population of these patients, the inclusion criteria do not encompass the duration of their non‐conception. Consequently, instead of the term “infertility,” which is internationally defined as non‐conception after 1 year of unprotected intercourse, the term “subfertility” was employed to more generally describe individuals suspected of having reduced fertility.^1^

### 2.2 Patient evaluation and pregnancy outcomes

In both segments of the study, semen samples were collected in sterile containers through masturbation and allowed to liquefy, and the aliquots were subsequently extracted for analysis. Parameters, including semen volume, sperm concentration, percent motility, total sperm count, and total motile sperm count, were assessed in accordance with WHO 2010 criteria.^19^, ^21^ However, following the publication of the WHO 2021 criteria during the study, these new standards were also applied. Specifically, in the second segment of our study, the parameters for all patients were compared with the reference values outlined in the WHO 2021 criteria. All participants were instructed to abstain from ejaculation for 48 h to 5 days before semen collection, mainly conducted in facility‐designated rooms. A few opting for home collection were advised to keep the sample around 36°C and submit it to the laboratory within an hour post‐ejaculation.

Blood samples were drawn for the endocrinological screening in the second segment of the study to investigate subfertility. This screening included the measurements of serum hormones as follows: luteinizing hormone (LH), follicle‐stimulating hormone (FSH), prolactin (PRL), testosterone, and estradiol (E2). These hormones were quantified using a chemiluminescent immunoassay. The reference ranges for LH, FSH, PRL, testosterone, and E2 were 0.79–5.72, 2.00–8.30 mIU/mL, 4.29–13.69, 1.31–8.71 ng/mL, and 14.6–48.8 pg/mL, respectively.

Additionally, 124 of the 245 patients enrolled in this study were successfully followed up for 1 year, according to their medical records (Figure S1). The follow‐up was conducted to determine whether their partners achieved clinical pregnancies. Clinical pregnancy was characterized by the presence of a gestational sac or fetal heartbeat, excluding biochemical pregnancies. Pregnancy outcome was further categorized as follows: pregnancies resulting from either spontaneous pregnancies, irrespective of ovarian induction, or those achieved through intrauterine insemination (IUI), were exclusively delineated from pregnancies resulting from fresh or frozen embryo transfer following in vitro fertilization or intracytoplasmic sperm injection treatments (IVF/ICSI), based on the methodology of a previous study conducted at the same institution.^22^

### 2.3 Analysis of TEs

SP samples were obtained by centrifuging a minimum of 0.5 mL of semen post‐liquefaction, whereas serum samples were derived from 6 mL of clotted intravenous blood. Both samples were centrifuged for 10 min at 1500 *g* (12 cm, 3340 rpm). Finally, SP and serum samples were stored at −80°C in a refrigerator before TE level assessment.

The concentrations of various TEs in both SP and serum samples were detected using a previously described method.^23^ The Agilent 7800 equipment (Agilent Technologies, USA) was used to operate the inductively coupled plasma mass spectrometry (ICP‐MS), which successfully measured the TEs, including lithium (Li), sodium (Na), magnesium (Mg), phosphorus (P), sulfur (S), potassium (K), calcium (Ca), manganese (Mn), iron (Fe), cobalt (Co), copper (Cu), Zn, arsenic (As), selenium (Se), rubidium (Rb), strontium (Sr), molybdenum (Mo), cesium (Cs), barium (Ba), and thallium (Tl). As a pretreatment for ICP‐MS, 50 μL of SP or serum sample was digested with 25 μL each of 61% nitric acid (Kanto Chemical, Japan) and 30% hydrogen peroxide solution (Kanto Chemical, Japan) in a polypropylene container for 16 h at 70°C; the final volume was adjusted to approximately 2.5 mL with ultrapure water (resistivity of ≥18 MΩ·cm). A mixture of standard solution (10 ppm) (XSTC‐622B, SPEX, USA) and a single standard solution (1000 ppm) (for P, Fujifilm Wako, Japan; excluding P, Kanto Chemical) were diluted with a 3% nitric acid solution to prepare the calibration curves for TEs. The linearity of the calibration curve was evaluated based on a correlation coefficient of ≥0.9998 for any of the 20 TEs. ICP‐MS system was used for analysis with high‐frequency output mode (1550 W), plasma gas flow rate of 15 L/min, and nebulizer gas flow rate of 1.05 L/min, as internal standard elements (Kanto Chemical, Japan) of beryllium, yttrium, rhodium, and tellurium were added to 50, 5, 1, and 50 μg/L, respectively.

### 2.4 Calculation and comparison of SP/serum ratio data

For each of the 20 TE, the ratio of concentration in SP to concentration in serum was calculated for the purpose of qualitative characteristic comparison and statistical analysis. For comparison of qualitative characteristics, the SP/serum ratio scale was used to extract variables of interest and visualize high/low patterns.^12^

### 2.5 Statistical analyses

In the first segment of the study, a paired *t*‐test was used to analyze the corresponding two‐group data for continuous variables. The second segment involved a detailed unsupervised cluster analysis using the ComplexHeatmap package (version 2.12.1) and the ConsensusClusterPlus package (version 1.60.0) in R,^24^ where various “k” values were tested to determine the most effective clusters. Subsequently, the k‐medoids method was used for precise clustering. Each cluster was analyzed using the Wilcoxon rank‐sum or Kruskal–Wallis test. Bonferroni correction was applied to address the multiple comparison problems. The chi‐square test was used to assess pregnancy outcomes, followed by the calculation of the standardized residual. Univariate and multivariate analyses were conducted to estimate the variables impacting pregnancy outcomes. The univariate analysis employed the Wilcoxon rank‐sum test for each semen quality parameter and the SP/serum ratio for each TE. For the multivariate analysis, logistic regression was performed with variables of interest, and three semen quality parameters—semen volume, sperm concentration, and motility—were selected for their low variance inflation factor to avoid multicollinearity. All statistical comparisons were two‐sided, and statistical significance was set at *p* < 0.05. All statistical analyses were performed using the R software (version 4.2.2; R Foundation, Vienna, Austria).

### 2.6 Ethical considerations

The University of Tsukuba Hospital, IUHW Hospital, and Renatech Corporation approved this study (approval numbers: University of Tsukuba: #H30‐152 and #R03‐127, IUHW: #13‐B‐358, and Renatech, #009). All participants provided written informed consent after the study's purpose, methods, potential benefits, and risks were thoroughly explained. The privacy and confidentiality of the participants were rigorously maintained throughout the data collection and analysis processes.

## 3 RESULTS

### 3.1 Analysis of specific differences in semen quality and SP/serum ratio of multiple TEs for each fraction of the ejaculation sample

**FIGURE 1:**
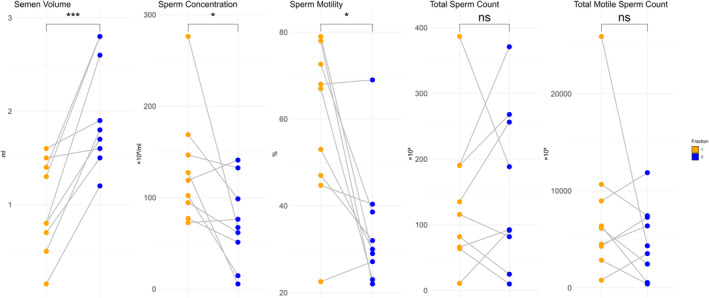
Comparison of sperm parameters among the nine sets of fractions of ejaculation samples. The five sets of dot plots show the semen volume, sperm concentration, motility, total sperm count, and total motile sperm count of fractions 1 and 2 in healthy volunteers. The orange and blue dots represent fractions 1 and 2, respectively, and a line connects the corresponding fractions. A paired t‐test for sperm parameters showed significant differences between fractions (*p < 0.05 and ***p < 0.001).

First, the corresponding nine pairs of fractions 1 and 2 in the healthy volunteer cohort were compared to determine the differences in the usual parameters of semen quality for each fraction of the ejaculation sample. The mean semen volume in fraction 2 was significantly higher than that in fraction 1 (0.97 vs. 1.99 mL, *p* = 0.0006) (Figure 1). Sperm concentration and motility in fraction 1 were significantly higher than those in fraction 2 (*p* = 0.038 and *p* = 0.012, respectively) (Figure 1). No significant difference was observed in either total sperm count or total motile sperm count between fractions 1 and 2 (Figure 1).

**FIGURE 2:**
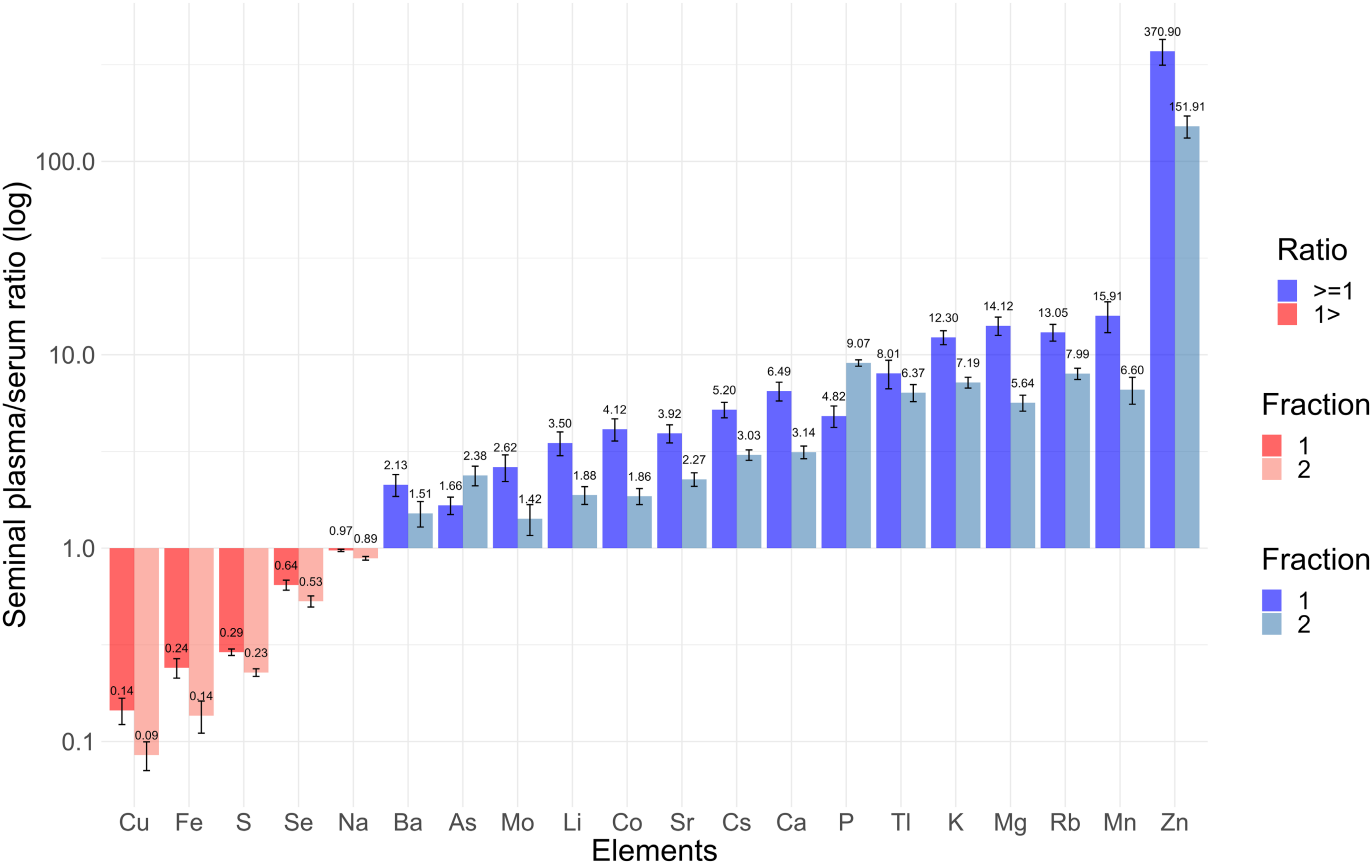
Scale patterns for the 20 TEs in the mean of SP/serum ratios across different fractions of ejaculation samples among healthy volunteers. The bar plot shows the mean SP/serum ratios of TEs among healthy volunteers, illustrating the scale patterns for 20 TEs and highlighting the differences between the fractions of ejaculation samples. The vertical axis represents the logarithmic scale in base 10. Elements with red and blue bars have a ratio of <1 and >1, respectively. The order of the bars ranges from fraction 1 to fraction 2 from left to right. SP, seminal plasma; TEs, trace elements.

Subsequently, the concentrations of 20 TEs in fractions 1 and 2 and the serum samples were measured for each participant to investigate the SP/serum ratio. As shown in Figure 2, the SP/serum ratios exhibited distinct scale patterns for the 20 TEs. Specifically, Cu, Fe, S, Se, and Na had ratios of <1, whereas the other 15 TEs had ratios of >1 in fractions 1 and 2. Notably, Zn had the highest ratio among the TEs. Furthermore, for comparison across different fractions of ejaculation samples, only two TEs, P and As, had significantly higher ratios in fraction 2 than in fraction 1 (*p* < 0.001 and *p* = 0.013, respectively). In contrast, the mean ratios of the other TEs in fraction 2 were lower than those in fraction 1 (Table S1). Therefore, these results suggest that the SP/serum ratio of multiple TE concentrations differs for each fraction of the ejaculation sample and the usual parameters of semen quality. Specifically, the relatively high SP/serum ratios of P and As and inversely high ratios of the other 18 TEs might be specific to fractions 2 and 1, respectively.

### 3.2 Differential distribution of the SP/serum ratios of TEs in male subfertility

Next, SP and serum samples collected from 245 patients were analyzed to investigate the SP/serum ratios of TEs in a real‐world male with subfertility population. Table 1 summarizes the patient characteristics. The median patient age was 35 years (interquartile range, 31–41 years). The median semen volume was 3.60 mL (interquartile range, 2.60–4.40 mL), and semen volume was higher than the reference value in 94.7% of patients. The median sperm concentration and motility were 39 × 10^6^/mL (interquartile range, 9–87 × 10^6^/mL) and 53% (interquartile range, 35%–68%), respectively. Sperm concentration and motility were higher than the reference value in 67.6% and 66.0% of the patients, respectively.

**TABLE 1:** Characteristics of the 245 patients.

Characteristic	*N* = 245^a^	WHO criteria	Meets criteria
Age (years)	35 (31, 41)		
Semen volume (mL)	3.60 (2.60, 4.40)	≥1.4	234 (94.7%)
Sperm concentration (10^6^/mL)	39 (9, 87)	≥16	167 (67.8%)
Sperm motility (%)	53 (35, 68)	≥42	163 (65.7%)
Total sperm count (10^6^)	127 (35, 321)	≥39	176 (71.0%)
Total motile sperm count (10^6^)	54 (12, 194)	Not applicable	

*Note*: Age was defined as the time of the initial visit. Semen volume, sperm concentration, sperm motility, and total sperm count were measured as previously described.

Abbreviation: WHO, World Health Organization.

^a^ Median [interquartile range (IQR)].

**FIGURE 3:**
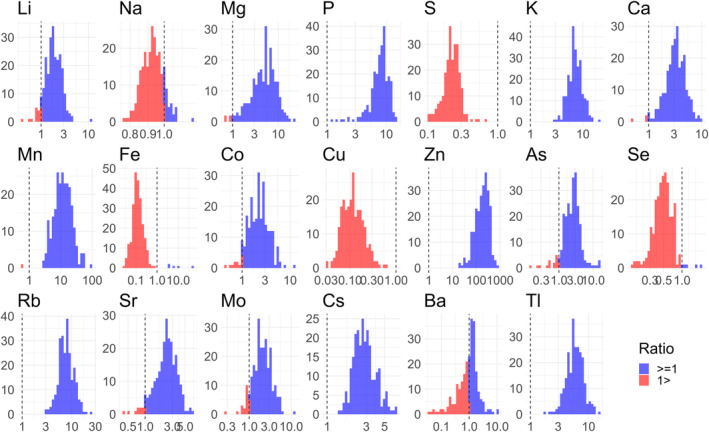
Distribution histograms of the SP/serum ratios for various TEs among the 245 patients. The 20 histograms show the SP/serum ratio of each TE among the 245 male patients with subfertility. The horizontal axis represents the logarithmic scale in base 10. The red and blue bars indicate a ratio of <1 and >1, respectively. SP, seminal plasma; TEs, trace elements.

Figure 3 illustrates the SP/serum ratio distribution histogram for the various TEs among the 245 patients. Most of the patients exhibited lower SP/serum ratios for TEs, including Na, S, Fe, Cu, and Se, indicating that their concentrations were lower in the SP than in the serum. Conversely, the number of patients with higher and lower concentrations of Ba in SP compared with serum was approximately equal. Almost all patients had higher concentrations of the other TEs in SP than in serum, with Zn being notably higher in the SP for many patients. However, a considerable patient‐to‐patient variation was found in these levels, suggesting variability within the male with subfertility population.

### 3.3 Potential subclassification of male subfertility based on the SP/serum ratios of multiple TEs

**FIGURE 4:**
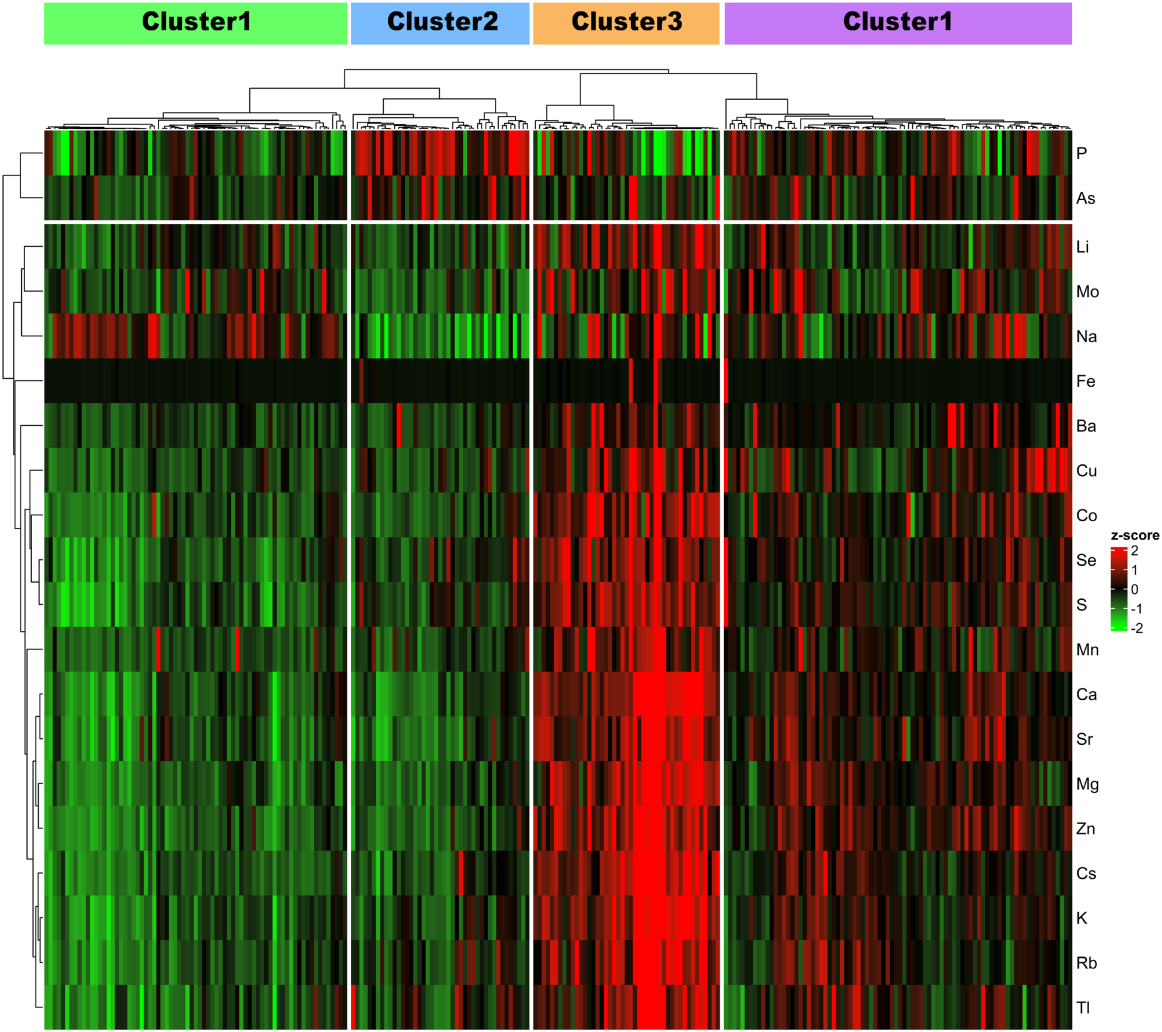
Consensus clustering heatmap of the SP/serum ratios of TEs. This heatmap shows the clustering results based on the SP/serum ratios of TEs from 245 male patients with subfertility. Rows represent the 20 TEs, and columns represent the SP/serum ratios of TEs in each patient. The color bar indicates that a high and low *Z*‐score is red and green, respectively. SP, seminal plasma; TEs, trace elements.

**FIGURE 5:**
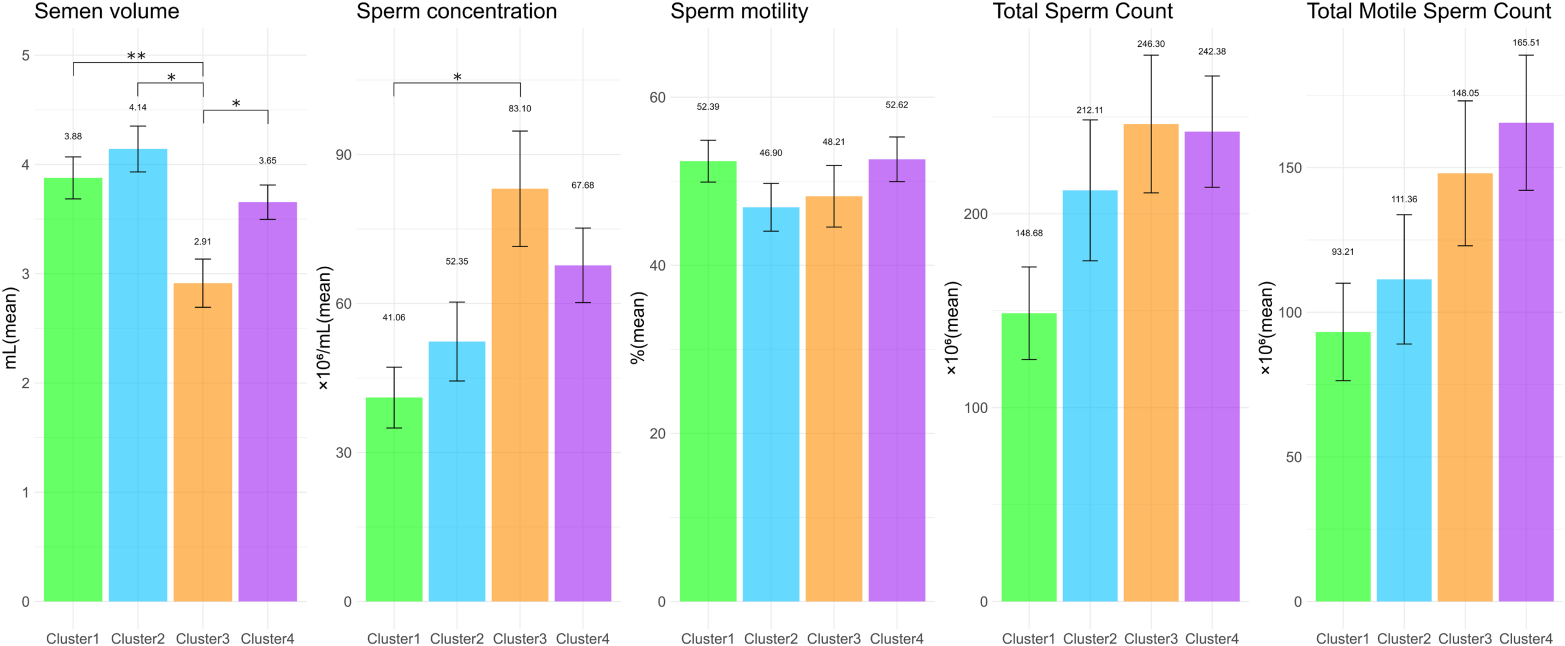
Features of semen quality parameters in each cluster. The five sets of bar plots show the semen volume, sperm concentration, motility, total sperm count, and total motile sperm count, respectively. They illustrate the features of these semen quality parameters across the clusters of 245 male patients with subfertility. A Wilcoxon rank‐sum test with Bonferroni correction for the parameters showed significant differences between the clusters (*p < 0.05 and **p < 0.01).

Next, unsupervised cluster analysis was applied to the SP/serum ratios of multiple TEs to explore the potential subclasses in the 245 patients. The cumulative distribution function plots at cluster number k = 4 showed an approximate maximum distribution of the consensus index, indicating maximum clustering stability (Figure S2). Hierarchical clustering heatmap and k‐medoid clustering identified four distinct clusters, each with unique profiles for these ratios (Figure 4). Significant age differences were observed among these clusters; however, the endocrinological profiles were similar (Table 2). Semen volume also significantly varied between clusters 1 and 3, 2 and 3, and 3 and 4 (Figure 5). Significant differences were found in sperm concentration between clusters 1 and 3 (Figure 5). No significant difference was observed in motility, total sperm count, or total motile sperm count among these clusters (Figure 5). Therefore, these findings indicate that the SP/serum ratios of multiple TEs can effectively categorize male patients with subfertility into distinct subclasses with varying semen qualities.

**TABLE 2:** Differences in age and endocrinological profiles in each cluster.

Characteristic	Cluster 1, *n* = 73^a^	Cluster 2, *n* = 43^a^	Cluster 3, *n* = 45^a^	Cluster 4, *n* = 84^a^	*p*‐Value^b^
Age, years	34 (30, 38)	36 (31, 39)	38 (34, 45)	34 (31, 41)	0.017
LH (mIU/mL)	2.49 (1.96, 3.46)	2.54 (1.95, 3.33)	3.02 (2.08, 3.66)	2.70 (2.02, 3.96)	0.5
FSH (mIU/mL)	4.0 (3.1, 5.8)	4.5 (3.1, 5.9)	5.0 (3.2, 7.0)	4.2 (2.8, 5.9)	0.4
PRL (ng/mL)	7.6 (5.8, 9.2)	8.7 (6.0, 11.9)	7.1 (6.0, 8.9)	8.7 (6.0, 10.6)	0.2
Testosterone (ng/mL)	4.44 (3.66, 5.10)	4.57 (3.46, 5.27)	4.60 (3.68, 5.72)	4.31 (3.66, 6.04)	0.9
E2 (pg/mL)	18 (12, 22)	19 (11, 22)	18 (12, 25)	19 (13, 24)	0.8

*Note*: The numbers listed represent the 25th, 50th, and 75th percentiles. The Kruskal–Wallis rank‐sum test for age showed significant differences between the clusters.

Abbreviations: E2, estradiol; FSH, follicle‐stimulating hormone; LH, luteinizing hormone; PRL, prolactin.

^a^ Median [interquartile range].

^b^ Kruskal–Wallis rank sum test.

### 3.4 Potential correlation between the SP/serum ratios of TEs in the subclasses of male patients with subfertility and the fractions of ejaculation samples

**FIGURE 6:**
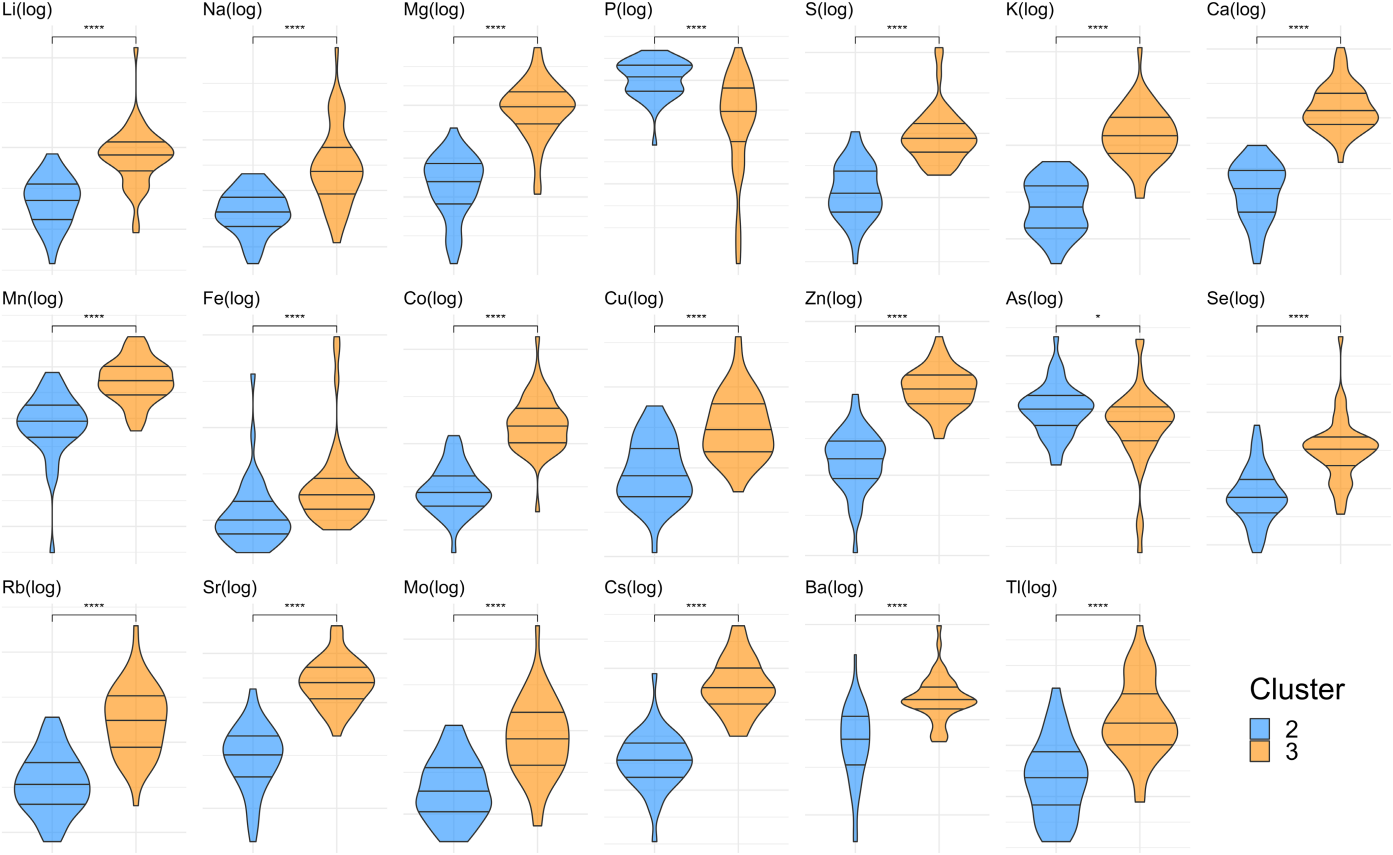
Comparison of the distributions of the SP/serum ratios of TEs between clusters 2 and 3. The 20 sets of violin plots show the comparison of the SP/serum ratio distributions for each TE between clusters 2 and 3, which were extracted from four clusters of 245 male patients with subfertility. The centerline denotes the median value (50th percentile), and the lines above and below denote the 25th–75th percentiles of the dataset. A Wilcoxon rank‐sum test for the means of the SP/serum ratios of TEs showed significant differences between the clusters (*p < 0.05 and ****p < 0.0001). SP, seminal plasma; TEs, trace elements.

Regarding the characteristics of the SP/serum ratios among the four clusters (Figure 4), hierarchical clustering clearly differentiated P and As from the other 18 TEs, as shown in the dendrogram. The clustering heatmap demonstrated a distinct distribution pattern as follows: P and As were predominantly found in cluster 2, whereas most of the other 18 TEs were detected in cluster 3. This pattern was also evident in the significant differences in the ratios of P and As compared with those in the other 18 TEs between clusters (Figure 6).

**FIGURE 7:**
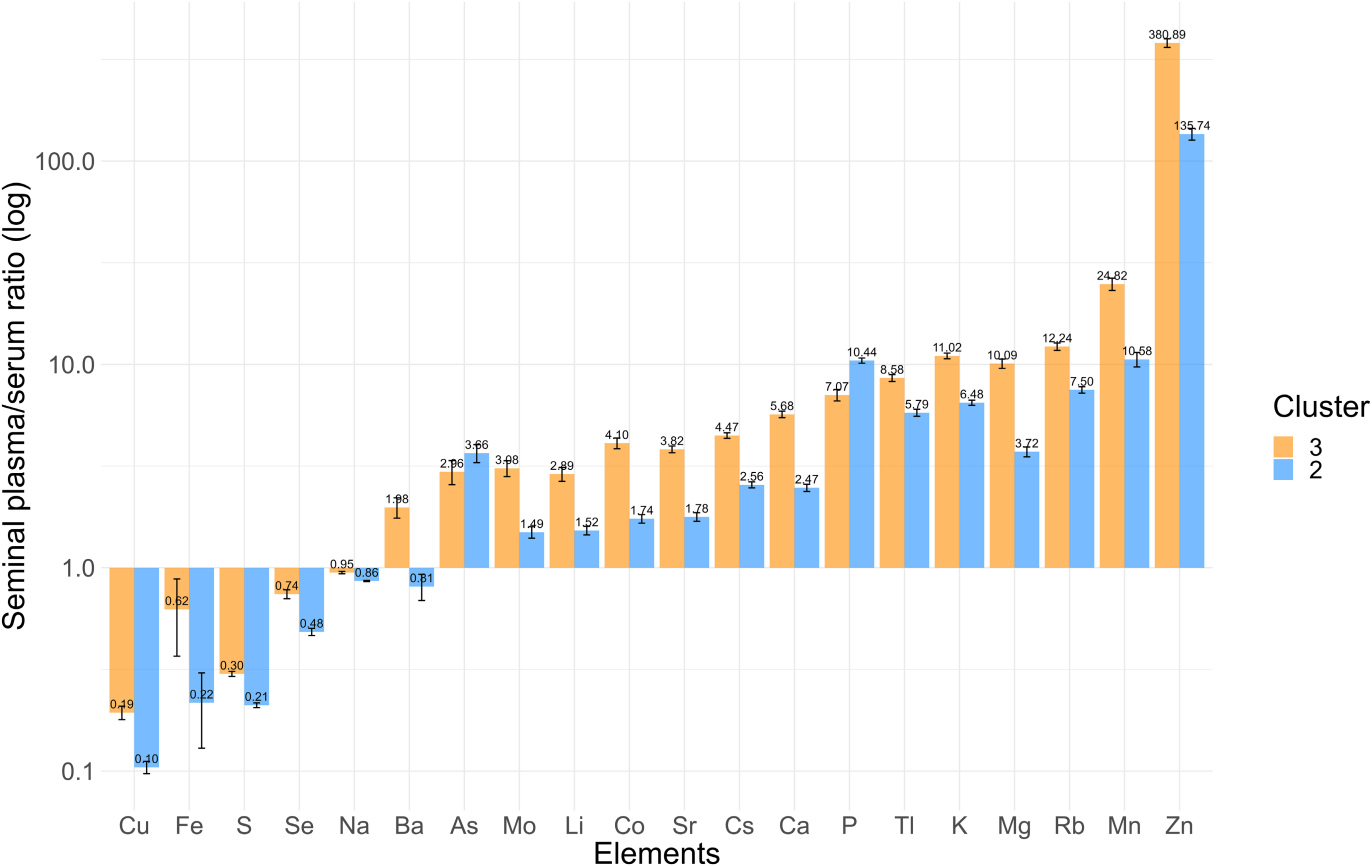
Scale patterns for the 20 TEs in the mean SP/serum ratios between clusters 2 and 3. The bar plot shows the mean SP/serum ratios of TEs among clusters of interest extracted from the four clusters of 245 male patients with subfertility, illustrating the scale patterns for 20 TEs and highlighting the differences between clusters 2 and 3. The vertical axis represents the logarithmic scale in base 10. The orange and blue bars represent clusters 3 and 2, respectively. SP, seminal plasma; TEs, trace elements.

Further investigation linked these findings to the different phases of ejaculation. Higher ratios of P and As may be associated with fraction 2 (subsequent phase), while the other 18 TEs were correlated with fraction 1 (early phase) (Figure 2). This correlation was explored in the healthy volunteer and male with subfertility cohorts. As shown in Figures 2 and 7, the scale patterns of SP/serum ratios for the 20 TEs in these cohorts were similar to each other; specifically, P and As were elevated in fraction 2 and cluster 2, while the other 18 TEs elevated in fraction 1 and cluster 3. Therefore, these results suggest a potential correlation between the SP/serum ratio in the subclasses of males with subfertility and fractions of ejaculation samples. Specifically, the subclasses with a high ratio of P and As and those with a high ratio of the other 18 TEs appeared to align with the different fractions of ejaculation samples.

### 3.5 SP/serum ratios in the subclasses of male patients with subfertility correlate with pregnancy outcomes

**FIGURE 8:**
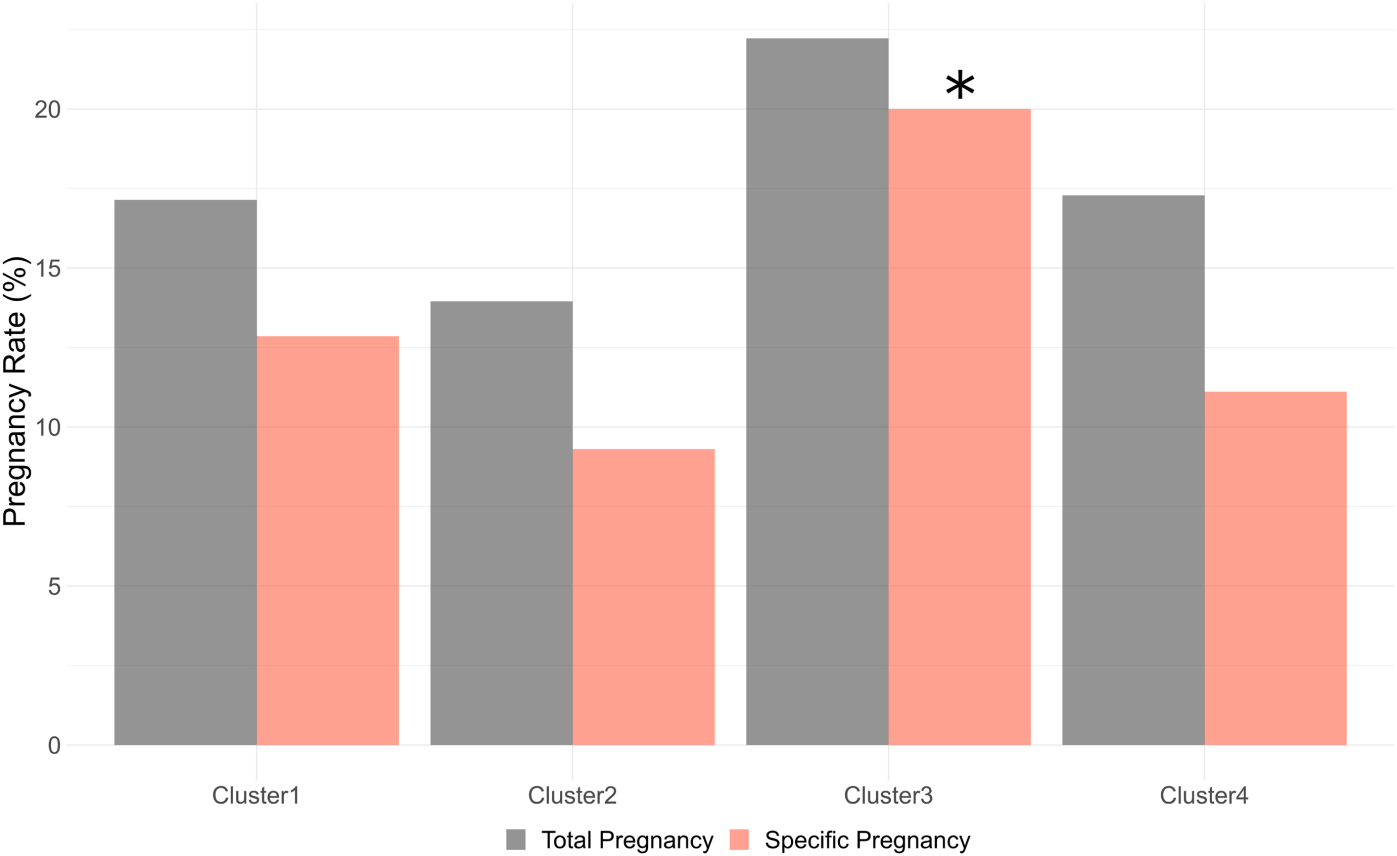
Pregnancy outcomes between each cluster. The gray and pink bars represent the total and specific pregnancy rates, respectively. Herein, specific pregnancy rates were defined as those exclusively resulting from either spontaneous pregnancy or intrauterine insemination. Analysis of the standardized residuals for the parameters showed significant differences (*p < 0.05).

The total pregnancy rates and specific pregnancy rates—the latter exclusively resulting from either spontaneous pregnancies or IUI—were briefly investigated for reference to examine the pregnancy outcomes for each of the four subclasses of male patients with subfertility. Although the chi‐square test failed to reject the null hypothesis of independence between the subclasses and pregnancy outcomes (total and specific pregnancy rates; *p* = 0.3867 and 0.1912, respectively), the analysis of standardized residuals indicated a significantly higher specific pregnancy rate in cluster 3 than in the other clusters (adjusted standardized residuals: 2.074; *p* = 0.0381) (Figure 8).

For supplementary analysis, 124 patients with known pregnancy outcomes were categorized into two groups: a more fertile group (*n* = 31) who achieved pregnancy through spontaneous pregnancies or IUI within a 1‐year follow‐up period and a less fertile group (*n* = 93) comprising the remainder. The remainder includes those who either achieved their first pregnancy through IVF/ICSI within the same 1‐year follow‐up period or did not achieve pregnancy at all, regardless of the fertility treatments attempted. Univariate analysis, employing each semen quality parameter and the SP/serum ratio for each TE, revealed that sperm concentration, motility, total sperm count, and total motile sperm count were significantly higher in the more fertile group (*p* = 0.027, 0.002, 0.037, and 0.013, respectively) (Table S2). However, no significant differences in the SP/serum ratio for any TE were found between the two groups (Table S2). Consequently, in the multivariate analysis, instead of individual TE's SP/serum ratios, results from cluster analysis that were statistically significant in previous analyses were introduced. This method suggested that being in cluster 3, alongside having motility above the WHO criteria of 42%, might be an independent predictor of higher fertility (Figure S3). Therefore, these findings indicate that the SP/serum ratios of multiple TEs could identify potential subclasses with superior pregnancy outcomes, which also correlates with the fractions of ejaculation samples.

## 4 DISCUSSION

This study, comprising two different segments, explored the association between TEs and the fractions of ejaculation samples and developed a new clustering model based on the SP/serum ratios of TEs. The first segment revealed significant differences in the TEs across the fractions from the split ejaculation sampling. Notably, P and As alone were higher, whereas most other TEs were lower in the fractions of the subsequent phases of ejaculation (Figure 2). This fraction also exhibited lower sperm concentration and motility (Figure 1). Furthermore, the second segment showed that the real‐world male with subfertility population can be classified into four clusters based on the SP/serum ratios of TEs, distinguishing between the early and subsequent phases of ejaculation without the split ejaculation sampling (Figure 4). This finding suggests that analyzing multiple TEs and their SP/serum ratios could help identify the subfertility patterns in males associated with post‐testicular factors, thereby providing innovative diagnostic strategies.

The fraction in the early phase of ejaculation is known to physiologically contain most of the sperm.^25^ Notably, our study confirmed this finding by showing higher sperm motility during the early phase (Figure 1). Two possible explanations for this finding exist.^10^ First, sperm initially emerging in the prostatic urethra gain motility through the epididymal tract and are ejected with prostatic secretion.^26^ Second, the subsequent phase, mainly seminal vesicular secretion, contains sperm motility inhibitors, including prostaglandins.^27^ Indeed, both the prostatic and seminal vesicular secretions are indispensable for male reproductive functions, as indicated by rodent studies.^28^ However, the dominance of prostatic secretions may enhance semen quality, as has been reported in various studies.^25^, ^27^, ^29^, ^30^, ^31^, ^32^, ^33^, ^34^ Therefore, understanding the optimum levels of these secretions for semen quality and related pathologies is crucial, although the comprehensive literature is limited.

Split ejaculation sampling reveals various prostate substances that are believed to positively affect sperm.^34^, ^35^, ^36^ Our study revealed that most of the TEs, except for P and As, were predominantly found in the early fraction of the ejaculation sample. However, this finding partly contradicts the previously anticipated positive contribution to sperm production. Regarding favorably maintaining sperm functions, essential TEs, including Zn, Ca, Mg, Cu, and Se, which are crucial for male fertility,^37^ are also primarily secreted by the prostate, as reported in previous studies.^11^, ^38^, ^39^, ^40^ Conversely, S, Fe, Co, and Mo are occasionally viewed as being detrimental to male fertility,^41^, ^42^, ^43^, ^44^ whereas Li, Ba, and Tl are considered toxic.^45^, ^46^, ^47^ Therefore, the presence of prostate‐dominant TEs does not necessarily have a beneficial effect on sperm. The prostate might not serve as a supplier of essential TEs to the SP alone but also as a discharge pathway for miscellaneous TEs, given its tendency to accumulate various TEs.^48^

In contrast, seminal vesicular secretion usually affects sperm negatively.^27^ This study found P and As to be the dominant TEs in seminal vesicles; however, their specific roles are unclear. Previous studies have linked P to seminal vesicles,^49^, ^50^ and excess P has been shown to harm male reproductive function through oxidative stress.^51^ Although As is known for its toxicity to male fertility,^52^ only a few studies have examined its relationship with seminal vesicular secretions. A study conducted in male mice indicated that As accumulates in the seminal vesicle relatively independent of the exposure levels compared within the prostate, where significant As accumulation requires exposure to high doses.^53^ Specifically, P and As may be exceptional TEs with a propensity to accumulate in seminal vesicles.

Although serum is known to contain various TEs,^37^, ^54^, ^55^, ^56^, ^57^ the available research is limited. Our study revealed extensive differences in TE levels between the SP and serum samples, as indicated by the SP/serum ratios. The levels of various TEs, most notably Zn, were maintained at higher concentrations in SP than in serum, whereas the levels of Cu, Fe, S, and Se were exclusively maintained at lower concentrations in SP than in serum (Figure 3). The varying SP/serum ratios of different substances imply intentional regulation by the epithelial cells in the accessory glands.^12^ Although the underlying physiological reasons for maintaining each TE at specific levels in the SP remain unclear, the multiplicity levels of the SP/serum ratios for each TE could strengthen its potential as a biomarker for assessing the secretory function of the accessory glands.

Estimating the fractions of ejaculation samples without split ejaculation sampling is challenging.^26^ Here, simultaneously collecting SP and serum from male patients with subfertility, measuring the concentrations of multiple TEs using ICP‐MS, calculating the SP/serum ratios, and clustering based on the K‐medoids method were designed to detect a specific population of TEs and discover the variations related to the accessory glands independent of split ejaculation sampling. Consequently, four characteristic clusters emerged, two of which were notably different (Figure 4). Cluster 2 displayed high P and As levels and low levels of the other TEs, suggesting a fraction of the subsequent phase of ejaculation, namely, seminal vesicular secretion dominance (Figure 4). Cluster 3 exhibited low P and As levels and high levels of the other TEs, indicating a fraction of the early phase of ejaculation, namely, prostatic secretion dominance (Figure 4). Furthermore, the corresponding scale patterns for the 20 TEs in the mean SP/serum ratios appeared to be similar (Figures 2 and 7). Interestingly, cluster 3 correlated with a slightly higher sperm concentration and an increased pregnancy rate despite significantly lower semen volumes (Figure 5), indicating prostatic secretion. Additionally, clusters 2 and 3, which are notable for their considerable size, may signify the dominance of fractions 2 and 1, respectively, indicating potentially unrecognized variations in male patients with subfertility, including those previously categorized as idiopathic. Specifically, this implies that unknown post‐testicular factors may contribute to the unexplained decline in semen quality.

This study has three notable advantages. First, this study used ICP‐MS for the simultaneous, cost‐effective, simple, and highly reproducible measurement of multiple TEs in different biological matrices.^58^ Although Zn and fructose have been sufficiently established as markers for evaluating the accessory glands,^59^, ^60^, ^61^ their assay systems are not widely available, and their clinical outcomes are under‐researched, leading to an undervaluation of SP biomarkers.^62^ Second, this study is almost the only approach to assess the function of the accessory glands by measuring TEs; even in the last few years, no similar studies exist among the many studies, mainly from India and China, that investigated the relationship between TEs as environmental pollution and male subfertility despite the advancement in various measurement techniques.^13^ In Japan, this study represents the first accomplishment in decades to examine TEs in a male with a subfertility population.^55^, ^63^, ^64^, ^65^ Third, this study applied unsupervised machine‐learning technology, specifically the K‐medoids method, which is known for its robustness against noise and outliers.^14^ The application of these techniques to TE patterns in subfertility in this study contributes to research on semen quality and pregnancy outcomes. Consequently, these findings create new possibilities for personalized medicine in male subfertility, and future research, such as TE supplementation therapy, is expected to advance the understanding of post‐testicular factors.

This study had some limitations. First, this study focused on fractions of ejaculation samples in a limited population with a small sample size; therefore, the possibility of exceptional populations cannot be ruled out. Second, this study was based on two cross‐sectional surveys. Therefore, data collection may be incomplete, and the quality of longitudinal data may vary. Finally, validation assessments were not conducted to confirm these results. In any case, caution should be exercised when generalizing these results. Furthermore, future studies with larger sample sizes and prospective study designs should be adopted for further validation.

In conclusion, the results of this study showed that clustering based on the SP/serum ratios of multiple TEs might identify the subclasses within the real‐world male with a subfertility population that may possess different characteristics. This study clarifies the role of variations in the secretion of accessory glands and ejaculation sequences in classifying the physiology of male subfertility and may lead to the development of new individualized modifications.

## FUNDING INFORMATION

This study was supported by the Japan Society for the Promotion of Science KAKENHI, Grant Numbers 23K15756 and 21K16737, and the Japan Science and Technology Agency, Grant Number JPMJPF2017.

## CONFLICT OF INTEREST STATEMENT

The authors declare no conflict of interest.

## ETHICAL APPROVAL

The University of Tsukuba Hospital, IUHW Hospital, and Renatech Corporation approved this study (approval numbers: University of Tsukuba: #H30‐152 and #R03‐127, IUHW: #13‐B‐358, and Renatech, #009). All participants provided written informed consent after the study's purpose, methods, potential benefits, and risks were thoroughly explained.

## HUMAN RIGHTS STATEMENTS AND INFORMED CONSENT

All procedures followed were in accordance with the ethical standards of the responsible committee on human experimentation (institutional and national) and with the Helsinki Declaration of 1964 and its later amendments. Informed consent was obtained from all patients for being included in the study.

## Supporting information


Figure S1.

Figure S2.

Figure S3.

Table S1.

Table S2.

## Data Availability

The data underpinning this article will be shared upon reasonable request from the corresponding author.
