# Correction to “A new clustering model based on the seminal plasma/serum ratios of multiple trace element concentrations in male patients with subfertility”

**DOI:** 10.1002/rmb2.12613

**Published:** 2024-10-08

Tanaka T, Kojo K, Nagumo Y, Ikeda A, Shimizu T, Fujimoto S, et al. A new clustering model based on the seminal plasma/serum ratios of multiple trace element concentrations in male patients with subfertility. *Reprod Med Biol*. 2024;23(1):e12584. doi: 10.1002/rmb2.12584.

In Figure 4, “Cluster 1” is shown twice. The label on the right should correctly read “Cluster 4,” as shown in the revised figure below.

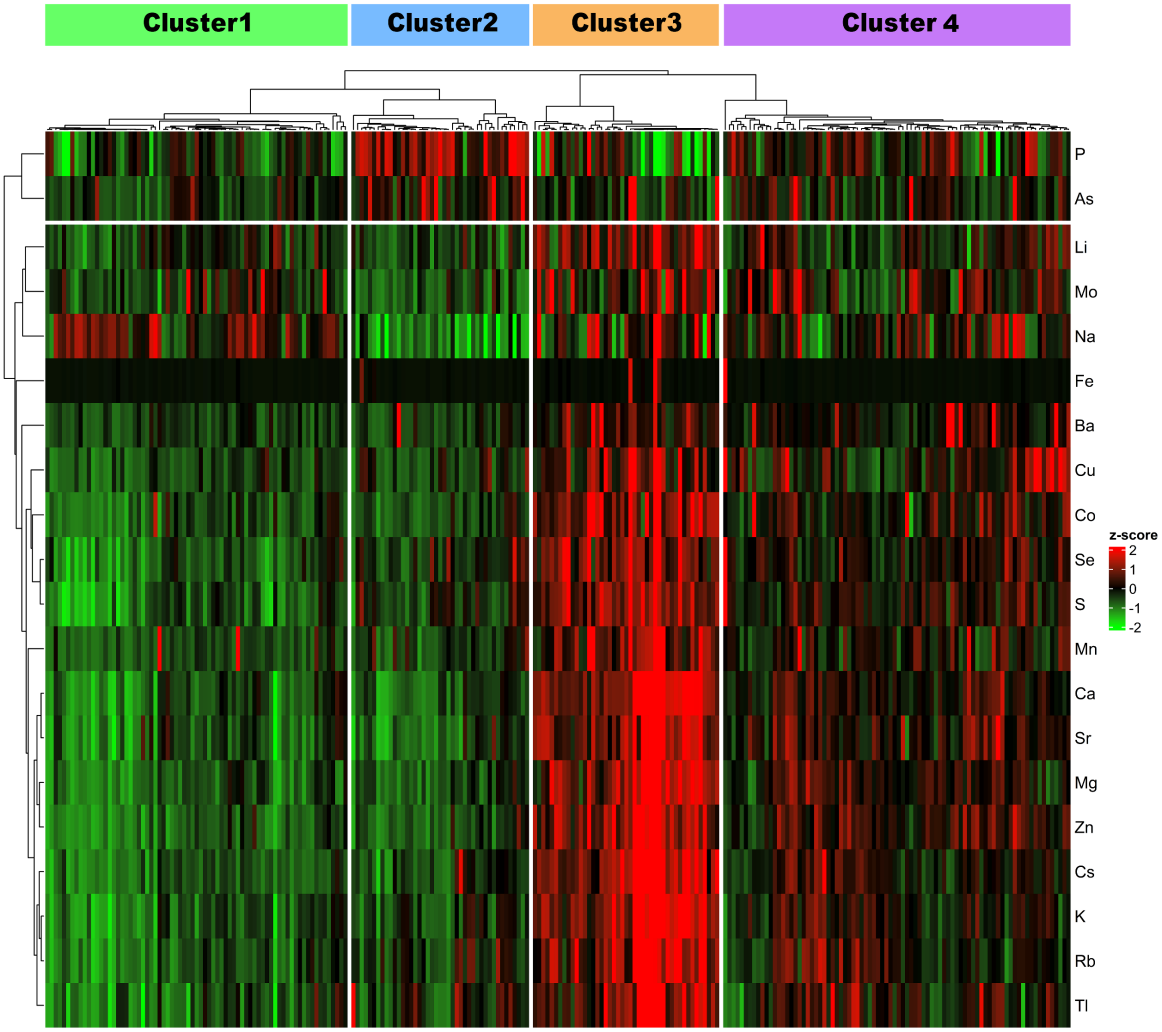

In Figure 8, eight bar graphs are displayed; however, they were drawn based on a different parameter from the cross‐tabulation analysis. The correct figure, drawn using the original values (sample proportions), includes error bars representing the values obtained by adding and subtracting the standard error. The corrected figure is provided below.

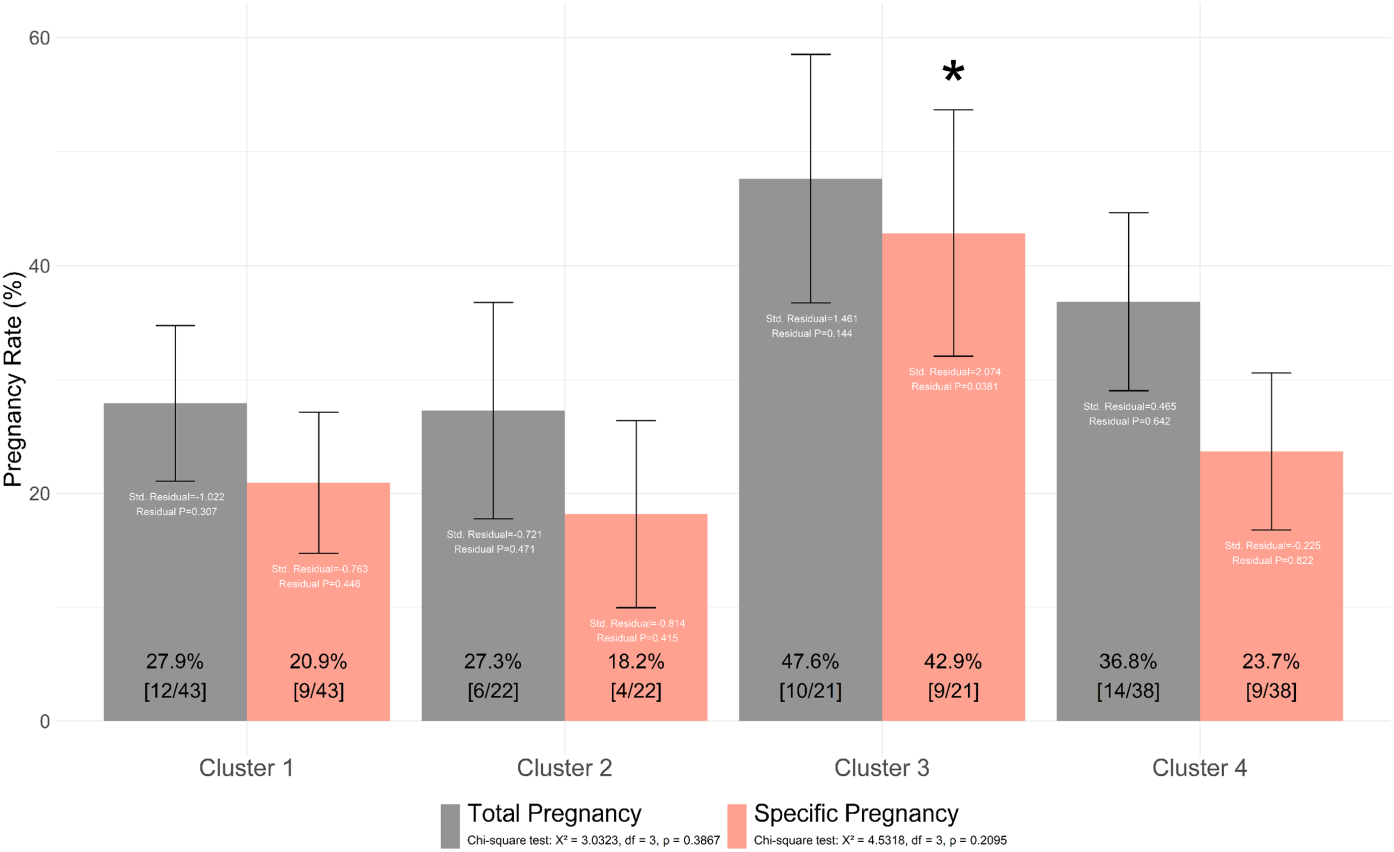

Additionally, concerning the error in Figure 8, the *p*‐value for specific pregnancy rates in the first paragraph of subsection 3.5 (SP/serum ratios in the subclasses of male patients with subfertility correlate with pregnancy outcomes) of the results section should be corrected to 0.2095.

We apologize for these errors.

