# Supplementary material for: A new clustering model based on the seminal plasma/serum ratios of multiple trace element concentrations in male patients with subfertility
Source: Reprod Med Biol. 2024 May 28;23(1):e12584. doi: 10.1002/rmb2.12584 (PMC11131575; doi:10.1002/rmb2.12584)
Supplement: Supplementary file 1 — Figure S1. [file RMB2-23-e12584-s005.pdf]

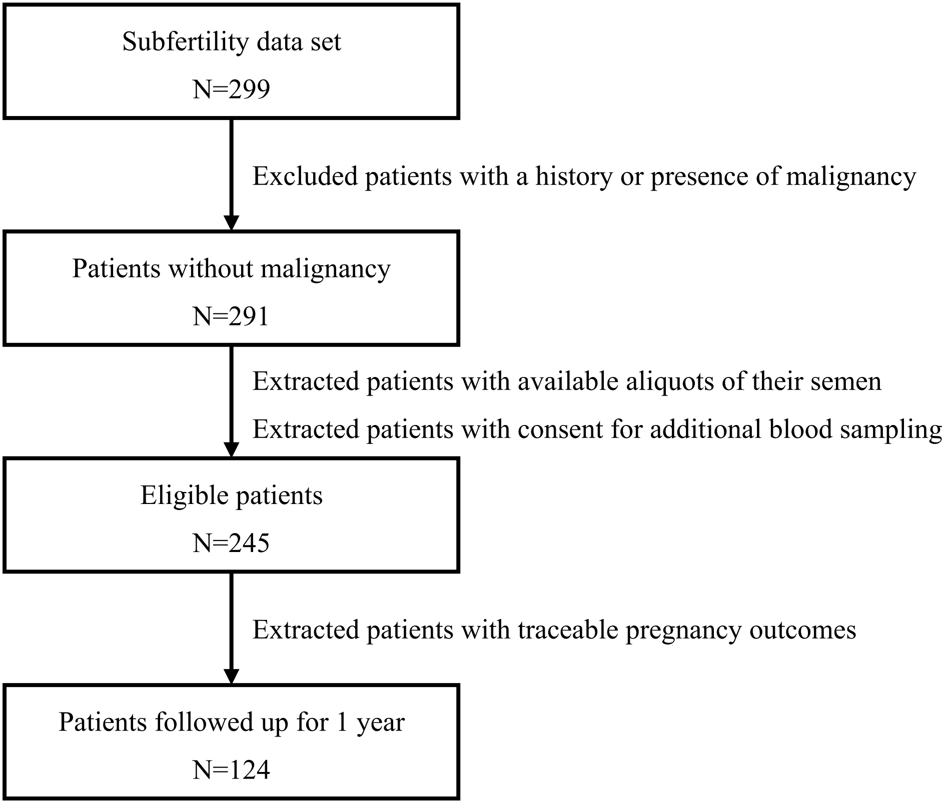

Figure S1: Participants selection for subfertility dataset.

Participants selection process for analysis. Participants with incomplete data obtained retrospectively from electronic medical records were excluded, as well as those with a history of malignancy. To further investigate the pregnancy rates, participants whose partners' pregnancy outcomes were known were selected.
