# Supplementary material for: A new clustering model based on the seminal plasma/serum ratios of multiple trace element concentrations in male patients with subfertility
Source: Reprod Med Biol. 2024 May 28;23(1):e12584. doi: 10.1002/rmb2.12584 (PMC11131575; doi:10.1002/rmb2.12584)
Supplement: Supplementary file 2 — Figure S2. [file RMB2-23-e12584-s004.pdf]

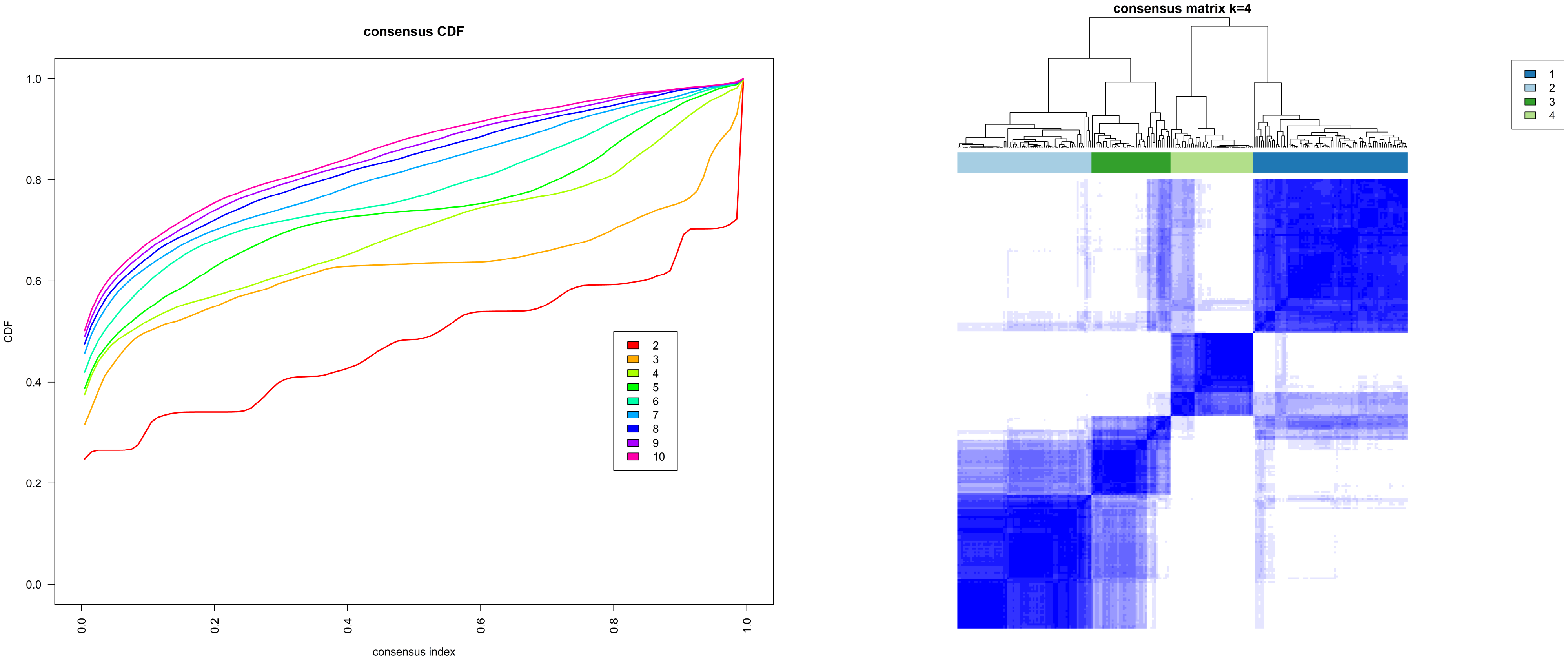

Figure S2: The cumulative distribution function (CDF) plot and consensus matrix.

Consensus matrices for cluster numbers at k=4 were analyzed using ConsensusClusterPlus with the SP/serum ratios of TEs.

TEs, trace elements: SP, seminal plasma
