# Supplementary material for: A new clustering model based on the seminal plasma/serum ratios of multiple trace element concentrations in male patients with subfertility
Source: Reprod Med Biol. 2024 May 28;23(1):e12584. doi: 10.1002/rmb2.12584 (PMC11131575; doi:10.1002/rmb2.12584)
Supplement: Supplementary file 3 — Figure S3. [file RMB2-23-e12584-s002.pdf]

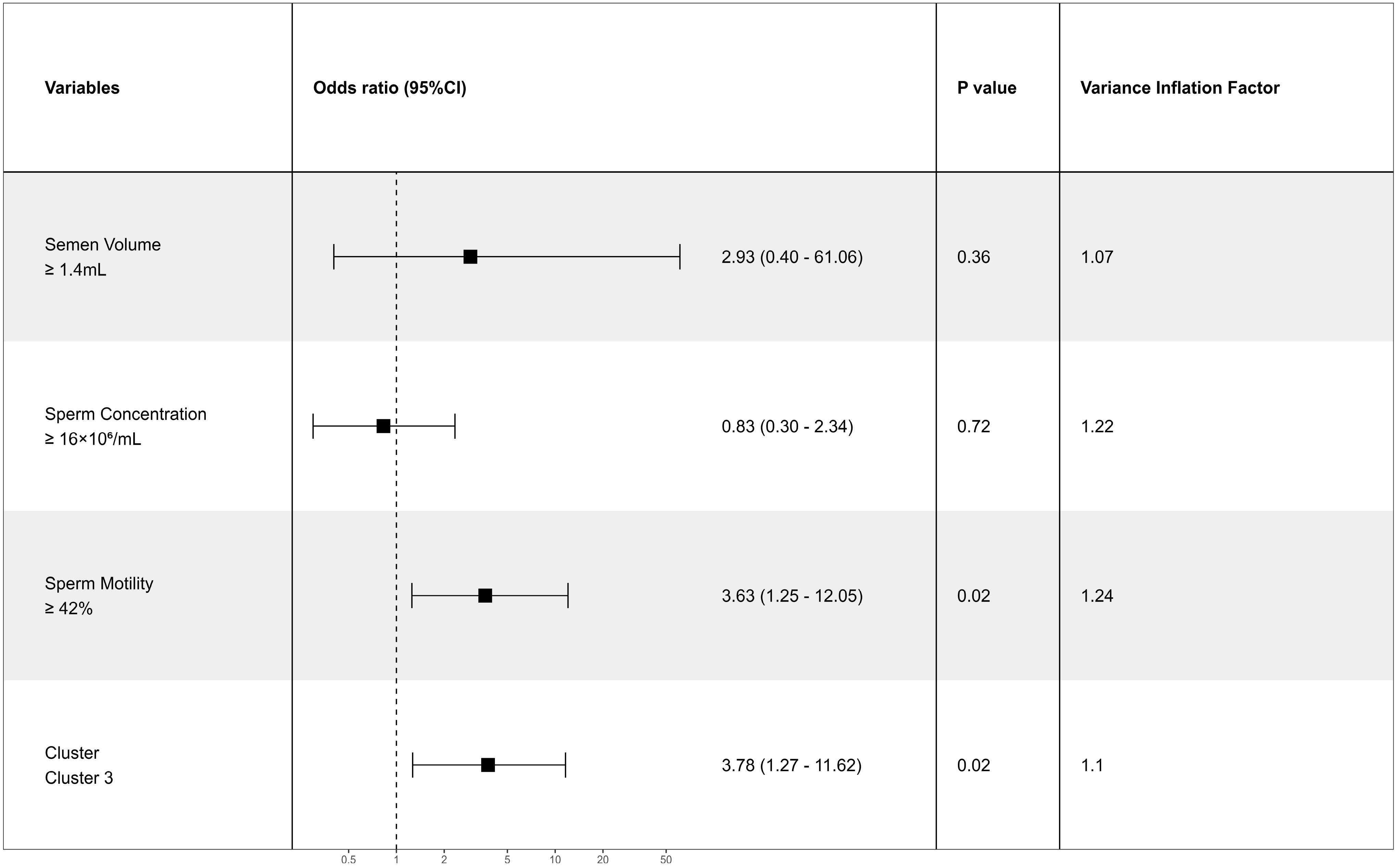

Figure S3: Multivariate analysis comparing pregnancy outcomes using representative parameters of semen quality and results from the cluster analysis.

The forest plot shows the effect on pregnancy outcomes. Overall, 124 patients with known pregnancy outcomes were categorized into two groups: a more fertile group (n=31) that achieved pregnancy through spontaneous pregnancies or intrauterine insemination within a one-year follow-up period and a less fertile group (n=93) comprising the remainder. The remainder includes those who either achieved their first pregnancy through *in vitro* fertilization or intracytoplasmic sperm injection within the same one-year follow-up period or did not achieve pregnancy at all, regardless of the fertility treatments attempted. Logistic regression was performed to determine odds ratios, 95%CIs, P-values, and variance inflation factor. The thresholds for each semen quality parameter subgroup were determined in accordance with WHO criteria. Clusters were categorized into cluster 3 and other than cluster 3. The solid boxes represent odds ratios, and the horizontal lines depict 95%CIs. The positioning of these variables to the right of the dashed line suggests their potential as independent predictors for being in the more fertile group.

95%CI, 95% confidence intervals
