# Supplementary material for: A new clustering model based on the seminal plasma/serum ratios of multiple trace element concentrations in male patients with subfertility
Source: Reprod Med Biol. 2024 May 28;23(1):e12584. doi: 10.1002/rmb2.12584 (PMC11131575; doi:10.1002/rmb2.12584)
Supplement: Supplementary file 4 — Table S1. [file RMB2-23-e12584-s003.pdf]

Table S1. **Mean SP/serum trace element ratios in each fraction**

**Characteristic**	**Fraction 1**, n = 9[1]	**Fraction 2**, n = 9[1]	p-value[2]
Li	3.50 (1.48)	1.88 (0.60)	**0.002**
Na	0.97 (0.04)	0.89 (0.06)	**<0.001**
Mg	14.1 (4.6)	5.6 (1.6)	**<0.001**
P	4.82 (1.83)	9.07 (1.04)	**<0.001**
S	0.29 (0.03)	0.23 (0.03)	**<0.001**
K	12.30 (3.07)	7.19 (1.38)	**<0.001**
Ca	6.49 (2.17)	3.14 (0.71)	**<0.001**
Mn	16 (9)	7 (3)	**0.006**
Fe	0.24 (0.08)	0.14 (0.08)	**0.002**
Co	4.12 (1.62)	1.86 (0.53)	**<0.001**
Cu	0.14 (0.07)	0.09 (0.04)	**0.010**
Zn	371 (169)	152 (60)	**<0.001**
As	1.66 (0.51)	2.38 (0.83)	**0.013**
Se	0.64 (0.11)	0.53 (0.11)	**0.007**
Rb	13.05 (3.88)	7.99 (1.60)	**<0.001**
Sr	3.92 (1.26)	2.27 (0.55)	**<0.001**
Mo	2.62 (1.24)	1.42 (0.77)	**0.009**
Cs	5.20 (1.43)	3.03 (0.57)	**<0.001**
Ba	2.13 (0.82)	1.51 (0.68)	**0.004**
Tl	8.01 (4.04)	6.37 (1.94)	0.086

[1]Mean (SD).

[2]Paired *t*-test.

The numbers listed represent the mean and standard deviation (SD). The paired *t*-test for the ratios showed significant differences between the fractions for 19 of the 20 elements.

Li, lithium; Na, sodium; Mg, magnesium; P, phosphorus; S, sulfur; K, potassium; Ca, calcium; Mn, manganese; Fe, iron; Co, cobalt; Cu, copper; Zn, As, arsenic; Se, selenium; Rb, rubidium; Sr, strontium; Mo, molybdenum; Cs, cesium; Ba, barium; Tl, thallium. SP, seminal plasma.
