# Supplementary material for: A new clustering model based on the seminal plasma/serum ratios of multiple trace element concentrations in male patients with subfertility
Source: Reprod Med Biol. 2024 May 28;23(1):e12584. doi: 10.1002/rmb2.12584 (PMC11131575; doi:10.1002/rmb2.12584)
Supplement: Supplementary file 5 — Table S2. [file RMB2-23-e12584-s001.pdf]

Table S2. Univariate analysis comparing pregnancy outcomes employing each semen quality parameter and the SP/serum ratio for each TE

Characteristic	More fertile, N = 31[1]	Less fertile, N = 93[1]	p-value[2]
Semen Volume (mL)	3.90 (2.80, 4.80)	3.60 (2.40, 4.30)	0.4
Sperm Concentration (10^6/mL)	67 (10, 110)	28 (6, 64)	0.027
Sperm Motility (%)	63 (50, 79)	46 (30, 64)	0.002
Total Sperm Count (106)	179 (48, 357)	94 (18, 232)	0.037
Total Motile Sperm Count (106)	104 (31, 291)	37 (5.0, 112)	0.013
Li	1.97 (1.42, 2.69)	1.88 (1.47, 2.38)	0.7
Na	0.94 (0.88, 0.97)	0.91 (0.88, 0.96)	0.5
Mg	5.3 (2.9, 7.3)	4.0 (2.8, 5.9)	0.2
P	7.07 (6.12, 9.24)	8.15 (6.22, 9.44)	0.5
S	0.23 (0.18, 0.26)	0.21 (0.18, 0.24)	0.4
K	6.81 (5.37, 9.00)	6.90 (5.69, 7.80)	>0.9
Ca	3.33 (2.18, 4.55)	3.14 (2.37, 3.72)	0.3
Mn	11 (7, 18)	10 (7, 15)	0.5
Fe	0.11 (0.08, 0.16)	0.11 (0.08, 0.17)	0.7
Co	2.11 (1.41, 3.14)	2.12 (1.52, 2.80)	>0.9
Cu	0.12 (0.08, 0.16)	0.10 (0.07, 0.17)	0.6
Zn	216 (116, 306)	162 (106, 265)	0.3
As	2.44 (1.61, 3.49)	2.52 (1.70, 3.14)	>0.9
Se	0.51 (0.37, 0.61)	0.47 (0.40, 0.59)	>0.9
Rb	7.57 (5.56, 8.83)	7.42 (5.96, 8.79)	0.8
Sr	2.59 (2.02, 3.62)	2.31 (1.74, 2.93)	0.1
Mo	2.18 (1.64, 2.95)	2.20 (1.57, 2.90)	0.7
Cs	2.86 (2.41, 3.51)	2.71 (2.36, 3.23)	0.4
Ba	1.36 (0.73, 1.55)	1.06 (0.51, 1.42)	0.14
Tl	5.55 (4.94, 6.92)	5.80 (4.62, 7.42)	0.7

[1]Median [interquartile range (IQR)]

[2]Wilcoxon rank sum test

The more fertile group was defined as those who, within a one-year follow-up period, had achieved pregnancy through spontaneous pregnancies or intrauterine insemination;
The less fertile group was defined as those who, within the same one-year follow-up period, either had achieved their first pregnancy through *in vitro* fertilization or intracytoplasmic sperm injection or had not achieved pregnancy at all, regardless of the fertility treatments attempted;
SP, seminal plasma; TE, trace element; Li, lithium; Na, sodium; Mg, magnesium; P, phosphorus; S, sulfur; K, potassium; Ca, calcium; Mn, manganese; Fe, iron; Co, cobalt; Cu, copper; Zn, zinc; As, arsenic; Se, selenium; Rb, rubidium; Sr, strontium; Mo, molybdenum; Cs, cesium; Ba, barium; Tl, thallium.
